# Targeted polar metabolomics data for *Neurospora crassa* wild type and mutant strains cultured on glucose

**DOI:** 10.1128/mra.01030-25

**Published:** 2025-11-06

**Authors:** Alexander J. Carillo, Lida Halilovic, Monique Quinn, Katherine A. Borkovich

**Affiliations:** 1 Department of Microbiology and Plant Pathology, University of California 207030, Riverside, California, USA; University of Maryland, Baltimore, Maryland, USA

**Keywords:** metabolomics, filamentous fungi, high-pressure liquid chromatography, mass spectrometry, polar metabolites

## Abstract

We present a metabolomics data set generated via high-performance liquid chromatography and high-resolution mass spectrometry for the filamentous fungus *Neurospora crassa* grown with glucose as the carbon source. A group of 121 polar primary metabolites was identified in wild type and gene replacement mutants lacking the heterotrimeric Gα subunits *gna-1* or *gna-3* or the non-receptor guanine nucleotide exchange factor *ric8*.

## ANNOUNCEMENT

We previously used ^1^H-nuclear magnetic resonance (^1^H-NMR) to profile metabolites from the filamentous fungus *Neurospora crassa* (1). However, ^1^H-NMR is limited by sensitivity (2), and liquid chromatography–mass spectrometry (LC–MS) is better for detection of metabolites in the picomole to femtomole range (3, 4). Here, we used LC–MS to identify relative levels of polar primary metabolites in four *N. crassa* strains (5), including wild type and three deletion mutants in which the coding region has been replaced with the *hph* selectable marker (6): the Δ*gna-1::hph* and Δ*gna-3::hph* Gα mutants and the non-receptor guanine nucleotide exchange factor mutant Δ*ric8::hph* (7–9). Strains (five biological replicates each) were inoculated to a density of 1 × 10^6^ cells/mL in Vogel’s minimal medium (10) containing 100 mM glucose as the carbon source. Cultures were incubated with shaking in the dark for 16 hours at 30°C, collected using vacuum filtration, and frozen. Cell pads were lyophilized at −80°C and dried samples were pulverized in a bead mill homogenizer. Samples containing 8–9 mg of tissue were combined with 100 µL of extraction buffer (acetonitrile:methanol:water:isopropanol [3:3:2:2]) per mg of tissue (11). The samples were sonicated and vortexed, followed by centrifugation at 16,000 × g for 15 min. The supernatant was stored at −80°C.

Liquid chromatography was performed using a ZIC-pHILIC column (2.1 × 150 mm, 5 µM) (EMD Millipore) and an I-Class UPLC (Waters). The mobile phases were (A) 5 mM ammonium bicarbonate in water, pH 9.6 and (B) 100% acetonitrile. The gradient was 0 min, 90% B; 1.5 min, 90% B; 16 min, 20% B; 18 min, 20% B; 20 min, 90% B; 28 min, 90% B. Mass spectrometry was accomplished using a TQ-XS triple quadrupole mass spectrometer (Waters), operated in selected reaction monitoring mode. All gases were nitrogen, except the argon collision gas. Source and desolvation temperatures were 150°C and 500°C, respectively. Desolvation gas was set to 1,000 L/h, cone gas to 150 L/h, and collision gas to 0.15 mL/min. Capillary voltage was 1 kV in positive ion mode and 2 kV in negative ion mode. A quality control sample, generated by pooling equal aliquots of each sample, was analyzed every 3–5 injections.

The Skyline for Small Molecules software package (accessed 15 Aug 2021) (MacCoss Lab Software) was used for raw data analysis (12). Default parameters were used for software. A transition list containing information on the 201 targeted polar metabolites, including retention time, ion mass ratios, and collision energy, was imported. Retention time peaks were standardized for each molecule. Each mutant was compared to the wild type to derive a relative abundance percentage. Standard error was calculated, and unpaired *t*-tests were used to determine statistical significance between strains (13).

**Fig 1:**
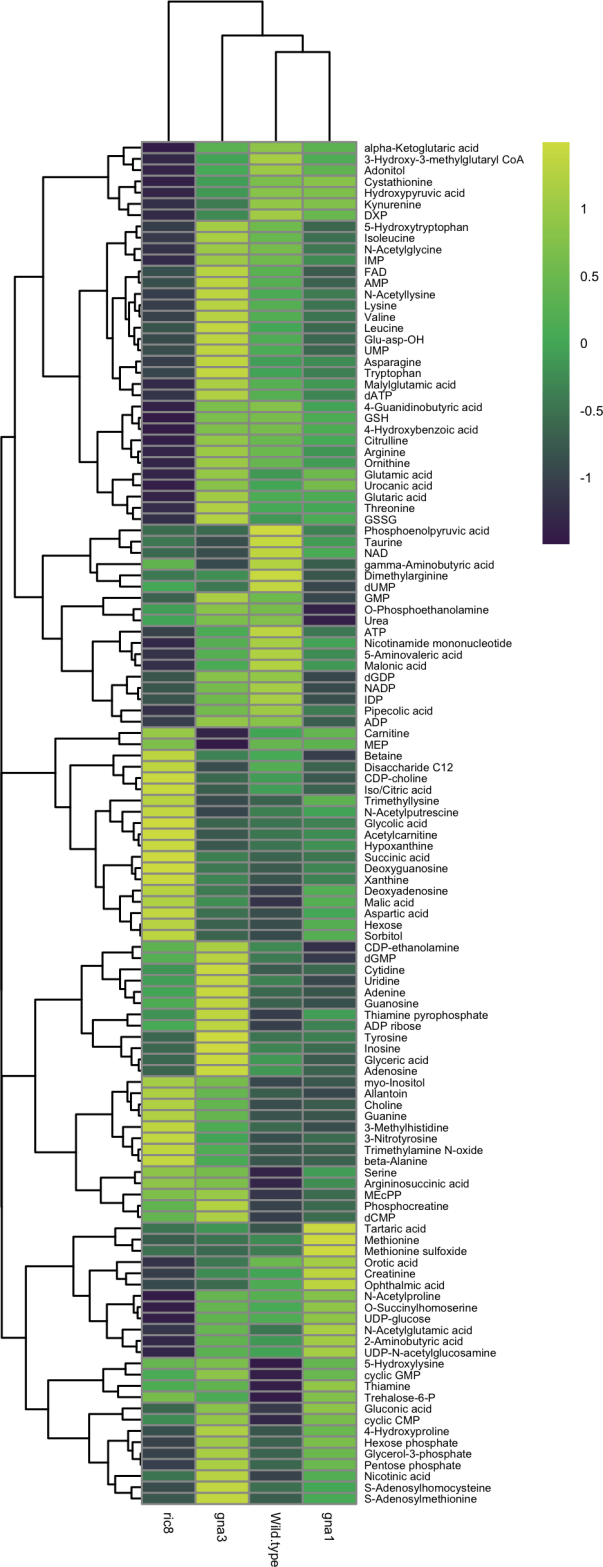
Hierarchical clustering to identify metabolites present at different relative levels in wild type and the three mutants during growth on glucose. Relative levels of 121 metabolites that could be detected in the four strains were used for clustering analysis and heat map generation. Yellow and green shading indicate higher and lower relative metabolite levels, respectively.

We were able to unambiguously detect and quantify 121 of the 201 (60%) primary polar metabolite standards in all four strains in the study (Fig. 1; Table 1). Four metabolites were present at levels higher than the threshold error and concentration. However, they were distinct spectra and were therefore detectable but not quantifiable (Table 1). Numerous differences in metabolite levels were noted between strains (Fig. 1), with the greatest number in the Δ*ric8::hph* mutant, including lower levels of 19/29 amino acids and several energy carriers (5). Our previous more limited ^1^H-NMR experiments (1) examined spectra for peaks corresponding to 102 metabolites, with only 21 unambiguously identified. Thus, there were numerous metabolites that were detected using LC–MS but not ^1^H-NMR, highlighting the relative advantage of using LC–MS for metabolomics experiments in *N. crassa*.

**TABLE 1:** Metabolite classes that were detected and quantified

Metabolite class	Total number of compounds^*a*^
Amino acid	29
Purine	21
Pyrimidine	9
Sugar, sugar phosphate, sugar alcohol	8
Organic acid	7
Acetyl amino acid/methyl amino acid	6
Electron carrier/energy	6
Oxidative stress	5
TCA cycle	4
Urea cycle	4
B vitamin	3
Methylation	3
Methylerythritol phosphate pathway (isoprenoid)	3
Phospholipid turnover	3
Carnitine	2
Glycolysis	2
Amine oxide	1
Cholesterol synthesis	1
Choline	1
Peptide	1
Phenolic	1
Polyamine	1
Total	121

^*a*^ Glycerophosphocholine, glutamine, phenylalanine, and proline were not included in these counts, as they could be detected, but not quantified.

## Data Availability

The full metabolomics dataset for every sample, including the raw chromatographic peak intensities for each identified metabolite, has been made publicly available in Figshare at the following link: dx.doi.org/10.6084/m9.figshare.30082936.
